# Treatment of Nonvariceal Gastrointestinal Hemorrhage by Transcatheter Embolization

**DOI:** 10.1155/2013/604328

**Published:** 2013-06-13

**Authors:** Muhammad Ali, Tanveer Ul Haq, Basit Salam, Madiha Beg, Raza Sayani, Muhammad Azeemuddin

**Affiliations:** Radiology Department, Aga Khan University Hospital, Stadium Road, P.O. Box 3500, Karachi 74800, Pakistan

## Abstract

*Purpose*. To investigate the sensitivity of mesenteric angiography, technical success of hemostasis, clinical success rate, and complications of transcatheter embolization for the treatment of acute nonvariceal gastrointestinal hemorrhage. *Material and Methods*. A retrospective review of 200 consecutive patients who underwent mesenteric arteriography for acute nonvariceal gastrointestinal hemorrhage between February 2004 and February 2011 was done. *Results*. Of 200 angiographic studies, 114 correctly revealed the bleeding site with mesenteric angiography. 47 (41%) patients had upper gastrointestinal hemorrhage and 67 (59%) patients had lower gastrointestinal hemorrhage. Out of these 114, in 112 patients (98%) technical success was achieved with immediate cessation of bleeding. 81 patients could be followed for one month. Clinical success was achieved in 72 out of these 81 patients (89%). Seven patients rebled. 2 patients developed bowel ischemia. Four patients underwent surgery for bowel ischemia or rebleeding. *Conclusion*. The use of therapeutic transcatheter embolization for treatment of acute gastrointestinal hemorrhage is highly successful and relatively safe with 98% technical success and 2.4% postembolization ischemia in our series. In 89% of cases it was definitive without any further intervention.

## 1. Introduction

Acute gastrointestinal (GI) hemorrhage is a commonly presenting medical emergency having a hospital mortality of around 10% [1]. Presentation may vary from insidious blood loss to potentially life-threatening hemorrhage [2]. The bleeding site determination is challenging as it involves entire gastrointestinal tract [2]. Upper gastrointestinal hemorrhage patients present with hematemesis or melena and the bleeding point is proximal to the ligament of Treitz, whereas gastrointestinal lower haemorrhage patients present with melena or hematochezia and bleeding point is distal to the ligament of Treitz [3–5]. Bleeding ceases spontaneously in approximately 75% of cases and can recur in 25% of cases, resulting in significant morbidity and mortality [6]. 

Therapeutic options available for patients with acute GI hemorrhage include conservative medical management, endoscopic coagulation, vasopressin infusion, therapeutic transcatheter embolization, and surgery [7, 8]. Endoscopy is considered as a first-line diagnostic and therapeutic procedure; its sensitivity reaches 100% in upper gastrointestinal bleed but in case of lower gastrointestinal bleed only probable bleeding source can be found (60% of cases). In stable patients, radionuclide and CT imaging plays a great role. Tc-99m RBC scintigraphy is more than 90% sensitive and specific in detecting a bleeding site anywhere in gastrointestinal tract. However, its limited resolution does not allow precise gastrointestinal bleed localization. 

Endoscopy can fail in approximately 32% of cases because of presence of stool, blood clots, and technical difficulties as time required for patient's preparation for colonoscopy [9]. In addition, bleeder source in small bowel is not accessible via colonoscope [10]. Significant morbidity and mortality are associated with emergency surgery [11]. Higher rates of complication and rebleeding were encountered in patients treated with vasopressin [12]. Nusbaum and Baum first described mesenteric angiography for acute GI hemorrhage in 1963 [13]. In 1972, Rösch et al. successfully controlled acute gastric hemorrhage by gastroepiploic artery embolization using autologous blood clot [14]. Due to significant technical improvements in the past 10 years selective therapeutic transcatheter embolization has become a safer procedure and is now widely used for acute GI hemorrhage management [15].

The purpose of our study was to investigate the sensitivity of mesenteric angiography, technical success of hemostasis, clinical success rate, and complications of therapeutic transcatheter embolization for the treatment of acute nonvariceal gastrointestinal hemorrhage. 

## 2. Materials and Methods

### 2.1. Study Group

We performed a single-center (from February 2004 to February 2011) retrospective survey of all patients in whom therapeutic transcatheter embolization was attempted for control of acute gastrointestinal bleeding. 

### 2.2. Patient Selection

All acute GI hemorrhage patients who underwent mesenteric angiograms during this period were enrolled. These patients had been referred to by abdominal surgeons, emergency room (ER) physicians, or gastroenterologists, and procedures were performed by experienced interventional radiologist. 

### 2.3. Clinical Data

The clinical and laboratory data, imaging and endoscopic findings, and care provided as well as the outcome data were obtained from medical records of our hospital. The following parameters were collected for each patient: age, gender, presenting symptoms, severity of hemorrhage, site of bleeding, comorbid, number of units of blood or packed red blood cells transfused, history of coagulation disorder, and findings of prior endoscopy or scintigraphy. Angiographic characteristics include segmental localization of bleeding in the gastrointestinal tract, vascular territory which is corresponding, catheters used for embolization, technique of embolization whether selective (proximal) or superselective (distal), repeat angiographic procedures, and type of embolic agent(s) used. Complications were divided into intraoperative and postprocedural complications. Follow-up duration as well as conservative or surgical management of complications was also documented.

### 2.4. Angiography and Embolization

Celiac, superior mesenteric, and inferior mesenteric angiography was performed transfemorally using 4 Fr or 5 Fr Cobra or Simmons type catheters (Cordis). Selective SMA angiography was also done by advancing catheter in different branches to evaluate the jejunal, ileal, ileocolic, and colic branches. Once the bleeding site was determined, then superselective catheterization was usually performed using a 2.7 Fr microcatheter (Progreat-Terumo), which is inserted coaxially through the macrocatheter. Superselective embolization was attempted by positioning the catheter as close to the bleeding site as possible. The materials used for embolization included microcoils (Cook and Balt), gelfoam, and polyvinyl alcohol particles (Boston Scientific). Other than active bleeding or pseudoaneurysm indirect sign for abnormal site was described when there were arteriovenous fistula vascular tuft, early filling vein, or a hyper vascular mass. In certain cases where there was endoscopic finding of active bleeding from duodenal region and a negative arteriography, a prophylactic embolization of the gastroduodenal artery was performed using Sandwich technique. In this the catheter is placed distal to the bleeding site followed by placement of coil, and then the catheter is withdrawn proximal to the bleeding site with deployment of another coil to sandwich the bleeding point in between. This technique ensures any retrograde filling of the targeted portion of the vessel embolized. In few cases with severe vasospasm intra-arterial nitroglycerine was also given.

All procedures were performed by vascular access at the level of the common femoral artery using 5 Fr vascular access sheath (Arrow, Medcomp). Postexamination, manual compression was maintained at puncture site with the patient lying in supine position for hemostasis.

### 2.5. Definition and Data Analysis

Successful embolization is termed when there is devascularization of a focal lesion or reduction or stoppage of flow to the vascular bed. We defined outcome criteria in accordance with the guidelines of the Society of Interventional Radiology (SIR) [16].

Technical success was described as immediate cessation of extravasation on postprocedure angiography [16].

Clinical success was described as nonoccurrence of bleed or hemodynamic instability within 30 days after embolization on follow-up evaluation. Monitoring was performed to evaluate signs and symptoms of intestinal infarction or ischemia [16].

Rebleeding was described as drop in hemoglobin >1 g/dL in the presence of overt GI hemorrhage within 30 days. An ischemic event was defined as bowel ischemia or infarction that required surgery [16].

Data was entered into SPSS statistical software version 19.0. Mean and standard deviations were computed for quantitative variables. Frequencies and descriptive analysis of the variables also measured. 

## 3. Results

From February 2004 until February 2011, a total of 200 patients underwent mesenteric angiography for acute GI hemorrhage at our institution. 114 patients (57%) had contrast blush or abnormal vascularity in the GI tract and underwent therapeutic transcatheter embolization.

There were 134 (67%) male and 66 (33%) female patients with male-to-female ratio of 2 : 1. Median age was 57 years (range: 8–97 years).

28 patients present with hematemesis, 66 with melena, and 106 with per-rectal bleeding. Severity of the symptoms was also calculated which were mild (*n* = 74), moderate (*n* = 96), and severe (*n* = 30). These are summarized in Table 1.

We also evaluated the effect of associated comorbid conditions which predispose to GI hemorrhage. 44  patients had no comorbid disease, 28 had infectious disease, 24 had chronic liver disease, 22 had hypertension, 15 had diabetes mellitus, 20 had chronic renal disease, 21 had malignancy, 10 had trauma, and 16 patients had miscellaneous comorbid diseases.

Blood was transfused in 145 (72.5%) out of 200 patients. Coagulation profile was deranged in 76 (38%) patients; the rest presented with a normal coagulation profile. 

Prior RBC-tagged scintigraphy was performed in 62 patients. In 49 (79%) it showed activity corresponding to bleeding site. Majority of these patients had lower GI haemorrhage. Endoscopy was also performed in 93 patients of which 66 (70.9%) were positive.

On mesenteric angiography 47 patients (41%) had upper gastrointestinal hemorrhage, whereas 67 (59%) had lower gastrointestinal hemorrhage.

Angiographically positive sites were stomach 10 (8.8%), duodenum 37 (32.5%), jejunum 9 (7.9%), ileum 10 (8.8%), caecum 29 (25.4%), ascending colon 8 (7.0%), transverse colon 1 (0.9%), descending colon 2 (1.8%), and rectosigmoid region 8 (7.0%).

Embolization was technically possible in 112 of 114 patients, reaching a technical success rate of 98.24%. In one patient technical failure was due to inability to catheterize the supplying artery. This patient had blunt abdominal trauma and presented with a large pseudoaneurysm filling from the inferior pancreaticoduodenal artery. After selective catheterization of the superior mesenteric artery multiple collateral vessels was seen at the origin of inferior pancreaticoduodenal artery with retrograde filling of gastroduodenal artery. Despite multiple attempts the collaterals supplying the pseudoaneurysm could not be catheterized. The second patient was a young woman who presented with per-rectal bleeding. Angiogram demonstrated an abnormal lesion in the rectum filling in the venous phase. There were multiple phleboliths suggesting venous hemangioma. Findings were discussed with referring surgeon and it was decided not to embolize this lesion due to low vascularity and risk of bowel ischemia. 

**Figure 1:**
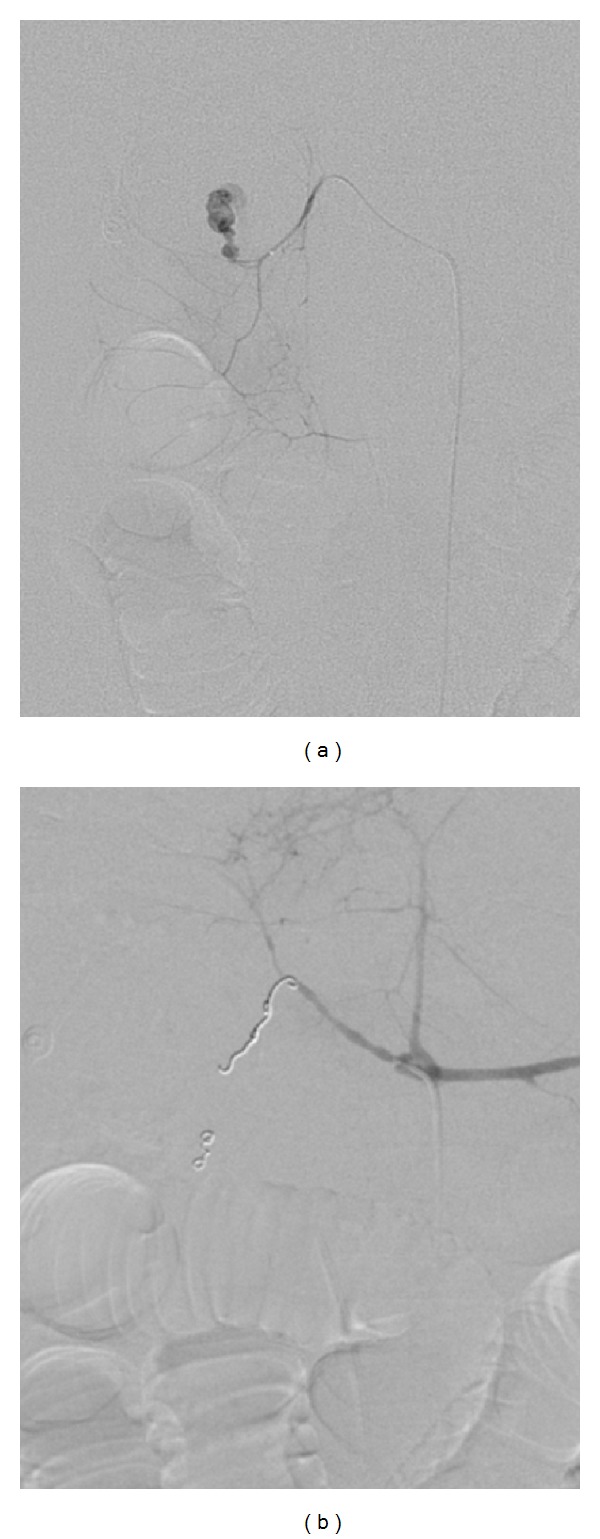
(a) Selective arteriogram of gastroduodenal artery demonstrates active bleeding from superior pancreaticoduodenal branch. (b) Postembolization arteriogram showed complete exclusion of the bleeding vessel by multiple platinum coils.

**Figure 2:**
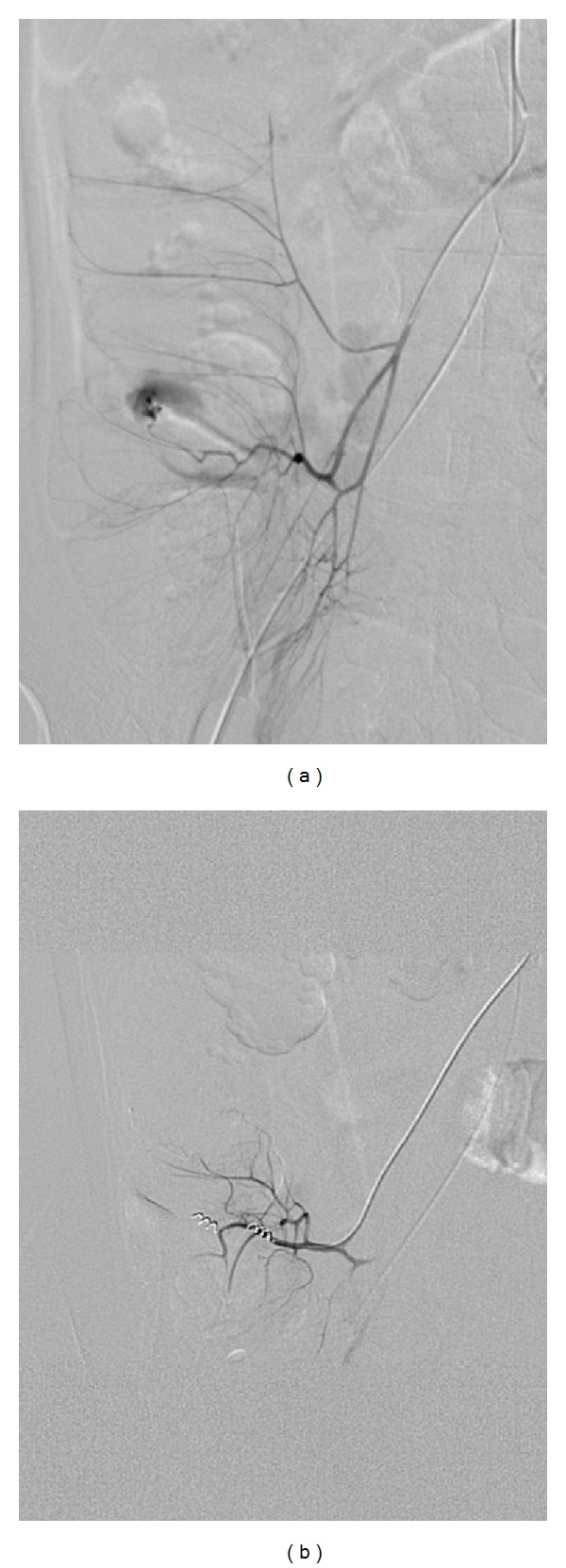
(a) Selective ileocolic artery angiogram demonstrates intraluminal extravasation of contrast in caecum. (b) Postembolization arteriogram after selective embolization shows total occlusion of feeding vessel and cessation of hemorrhaging.

Arteries primarily embolized were the gastroduodenal artery (*n* = 36) Figure 1, ileocolic artery (*n* = 28) Figure 2, right colic artery (*n* = 12), jejunal branches of superior mesenteric artery (*n* = 10), left gastric artery (*n* = 9), superior rectal artery (*n* = 7), ileal braches of superior mesenteric artery (*n* = 5), left colic artery (*n* = 4), and middle colic artery (*n* = 1).

We also evaluated the catheters used for angiography. In 109 (54.5%) patients microcatheter was used, whereas in 91 (45.4%) angiography was performed with regular 4 Fr catheters. Cobra catheter was used in 174 patients while Simmons catheter was used in 26 patients.

The type of embolic material used was at the discretion of the interventional radiologist, and in few patients materials were used in combination. Microcoils were used in isolation in 69 patients (62%) and in combination with particles in 9 patients (8%). Particles were used in isolation in 33 patients (29%) and gel foam was used in 1 patient (0.9%).

Out of 114 patients 81 were followed up with for one month, 17 for 1 week, and 1 for 1 day while 15 patients were lost to followup. Hence clinical success was measured in only 81 patients.

Total clinical success in the 30-day followup (i.e., complete resolution of signs or symptoms that prompted the embolization procedure) was achieved in 72 of 81 patients (89%). Seven out of 81 patients (11.5%) experienced clinical signs of early rebleeding Table 2. Corresponding vessels were gastroduodenal artery (*n* = 3), Jejunal branches of SMA (*n* = 2), and a single case from right ileocolic and left gastric arteries each. In 6 of them repeat angiographies were done, but only one patient showed recurrent jejunal hemorrhage and underwent clinically successful repeat embolization. 

The only major complication was early bowel ischemia experienced by 2 (2.4%) of the 81 patients. An elderly male was presented with perrectal bleeding. Arteriography showed abnormal vascularity in the territory of superior rectal artery which was partially embolized using PVA particles. He developed postembolization ischemia along with thrombosis of right common iliac vein. He underwent urgent rectosigmoid resection, while limb ischemia was successfully managed conservatively with oral anticoagulants. Second patient was an elderly male with bleeding duodenal ulcer on endoscopy. His empiric embolization of gastroduodenal artery was done. He presented with symptoms of ischemia after three days and was managed conservatively (Table 3).

After embolization 4 patients underwent surgery 3 for recurrent bleeding and 1 for ischemia. No patient died as a consequence of complications caused by the procedure. 

## 4. Discussion

Our data supports the present literature in demonstrating the efficiency of therapeutic transcatheter embolization in curing acute GI bleeding [5, 17]. In a recently published international consensus recommendation, arterial embolization is considered as a surgical alternative in upper GI bleeding management in whom endoscopic haemostatic procedure has failed or who had recurrent bleeding [16]. Similarly arterial embolization is now considered a first-line therapy for patients with severe lower GI bleeding [18].

The first objective of arteriography is to identify the bleeding site which requires the patient to be actively bleeding [19, 20]. In few patients even a detailed workup may fail to identify an exact bleeding site, which resulted in the patients undergoing repeated blood transfusions and invasive investigations [20]. In our study 57% of the angiographies were positive. Charbonnet et al. [2] reported a positive rate of 37% while survey by Zuckerman and Prakash [9] found that the rate of positive angiograms can vary from 27% to 77%. Chevallier and colleagues [21] reported very high percentage (93.4%) of positive angiographies which can be explained by the increased frequency of superselective catheter placement in these patients. Nevertheless an angiography may give normal results despite superselective catheterization because even a massive hemorrhage can be intermittent [4]. In these cases a blind or empirical embolization can be done, however, it is associated with an increased chance of rebleeding and ischemia [5, 7, 8]. To increase the positive rate prior to endoscopy, Tc 99 RBC-tagged scintigraphic evaluation or contrast enhanced CT scans could be helpful in identifying the bleeding vessel [6, 10, 20]. 

In our study prior Tc 99 RBC-tagged scintigraphy was performed in 62 patients with acute GI hemorrhage of which 49 (79%) were positive and helped in the identification of bleeding site. Similarly, endoscopy was performed in 93 patients of whom 66 (66%) were positive and guide towards the bleeding vessel.

Endoscopy for the management of gastrointestinal bleed still remains a feasible option whenever possible. Endoscopy can be diagnostic as well as therapeutic especially in upper GI bleed; however, in cases with massive GI bleed visualization becomes challenging and technically difficult. For lower GI bleed the technical success of endoscopy also depends on bowel preparation for optimal visualization. Evaluation of small bowel loops with endoscopy is not possible. For those patients in whom endoscopy was inconclusive or failed due to reasons described therapeutic transcatheter embolization is considered useful option. Surgery is rarely used for treatment of upper GI bleeds; however, it is still utilized for management of lower GI bleeds where endoscopic and transcatheter embolizations have failed or were not available. MDCT angio is considered as the initial radiological investigation as it is a very simple technique. It still requires active bleeding at the time of imaging but may be repeated in case of the first negative examination. A positive MDCT angio can select appropriate patients for rapid targeted embolization. The visualization of active extravasation of IV contrast in the GI tract requires careful attention to technique, including thin collimation, rapid IV contrast administration, and appropriate scan timing. The addition of multiplanar reconstructions (MPR) and 3D imaging is beneficial in identifying the exact source of the bleeding.

The present review, with a technical success rate of 98%, is concurrent with many other reports quoting technical success of more than 90% [4, 5, 8]. Defreyne and colleagues [22] reported technical success rate of 98%, while in a recent study Tan et al. [4] reported a technical success of 97%. In the literature only few technical failures have been reported, and they were mainly due to difficult vascular anatomy, vascular stenosis, and vascular spasms [15, 19]. In few cases intermittent contrast material extravasation can occur which reflects spontaneous bleeding arrests [22]. In such cases, the main arterial trunk should be vigorously studied to determine the point for safe embolization [3, 15, 22]. Because active extravasation was initially localized, we did not find indication for the use of anticoagulants or fibrinolytics, as recommended by some authors [22, 23].

There is a certain risk of complications with reported rates of major ischemic complications range from 0% to 16% [23, 24]. Very low rate (2.4%) of bowel ischemia in our study indicates that we delivered the right amount of embolic material in most of the cases. Luc Defreyne in 2001 observed absence of bowel ischemia in 40 patients [22]. Aina and colleagues [24] reviewed 75 consecutive patients who underwent arterial embolization for upper GI bleeding and reported a 99% technical success with a primary clinical success rate of 76%. Only three cases (4%) of ischemia were noted, two involving the duodenum and one the liver [24]. In our study rate of rebleeding was 8.6%. The reported rebleeding rate after therapeutic transcatheter embolization is approximately 33% (range: 9%–66%) [4]. Kwak et al. [5] found rebleeding in 21% of the patients while Wong and colleagues [11] reported high rate of rebleeding (34.4%). A possible explanation for the high rebleeding rate may be coiling the gastroduodenal artery from the celiac axis in these series as gastroduodenal artery can be later fed with collateral branches from the superior mesenteric artery [1, 11]. Our study also concurred with these results as 5 (38%) of the patients showed rebleeding from gastroduodenal artery. A sandwich technique can be used in these cases in which the gastroduodenal artery was coiled in a distal-to-proximal manner [1, 15]. Similarly inflammatory or ischemic reactions after embolization can trigger vasodilatation of the intramural collaterals and early rebleeding [12].

Our study had certain limitations, including the fact that it was a retrospective analysis, and the refinement and clinical validation of angiographic embolization require a prospective study. Secondly series of patients were enrolled from a single institution, and the cause of bleeding was not known in all cases.

In conclusion, therapeutic transcatheter embolization for the treatment of acute gastrointestinal hemorrhage is highly successful and relatively safe procedure with high technical and clinical success rates, and it should be reserved as a treatment option for patients who are high risk for surgery and failed endoscopic and medical management.

## Figures and Tables

**Table 1:** Demographics and characteristics of 200 patients who had mesenteric angiogram for acute GI hemorrhage.

Characteristics	Results
Angiographic sensitivity	114 (57%)
Median age	55 years
Gender	
Male	134 (76%)
Female	66 (33%)
Presenting complaints	
Hematemesis	28 (14%)
Melena	66 (33%)
Perrectal bleeding	106 (53%)
Prior investigation	
Upper GI endoscopy	93
Positive	66 (71%)
Negative	27 (29%)
RBC-tagged scintigraphy	62
Positive	49 (79%)
Negative	13 (21%)
Prior blood transfusion	145 (72.5%)
Coagulation profile	
Normal	124 (62%)
Deranged	76 (38%)
Comorbid	
No comorbid	44 (22%)
Chronic liver disease	24 (12%)
Hypertension	22 (11%)
Diabetes mellitus	15 (7.5%)
Chronic renal failure	20 (10%)
Malignancy	21 (10.5%)
Trauma	10 (5.0%)
Infectious diseases	28 (14%)
Misc	16 (8.0%)

**Table 2:** Characteristics of 81 patients after one-month followup.

Characteristics	Results
Clinical success	72 (89%)
Complication (bowel ischemia)	02 (2.4%)
Rebleeding	07 (8.6%)
Repeat procedure	06 (7.4%)
Surgical management	04 (4.9%)

**Table 3:** Characteristics of 114 patients who revealed bleeding site and underwent therapeutic embolization.

Characteristics	Results
Technical success	112 (98%)
Site	
Upper GI hemorrhage	47 (41%)
Lower GI hemorrhage	67 (59%)
Embolization	
Proximal (selective)	21 (19%)
Distal (superselective)	91 (81%)
Arteries embolized	
Gastroduodenal artery	36 (32%)
Ileocolic artery	28 (25%)
Right colic artery	12 (11%)
Jejunal branches of SMA	10 (8.9%)
Left gastric artery	09 (8.0%)
Superior rectal artery	07 (6.2%)
Ileal branches of SMA	05 (4.4%)
Left colic artery	04 (3.6%)
Middle colic artery	01 (0.9%)
Embolization material	
Microcoils	69 (62%)
PVA particles	33 (29%)
PVA with microcoils	09 (8.0%)
Gel foam	01 (0.9%)
